# Sensitizing tumor cells to conventional drugs: HSP70 chaperone inhibitors, their selection and application in cancer models

**DOI:** 10.1038/s41419-017-0160-y

**Published:** 2018-01-18

**Authors:** Vladimir F. Lazarev, Dmitry V. Sverchinsky, Elena R. Mikhaylova, Pavel I. Semenyuk, Elena Y. Komarova, Sergey A. Niskanen, Alina D. Nikotina, Anton V. Burakov, Viktor G. Kartsev, Irina V. Guzhova, Boris A. Margulis

**Affiliations:** 1 Laboratory of Cell Protection Mechanisms, Institute of Cytology of Russian Academy of Sciences, Tikhoretsky ave, 4, St. Petersburg, 194094 Russia; 2 A. N. Belozersky Research Institute of Physico-Chemical Biology, Moscow State University, Leninskye gory, house 1, building 40, Moscow, 119992 Russia; 3 InterBioscreen, Chernogolovka, Institutsky Ave, 7a, Chernogolovka, 142432 Russia

## Abstract

Hsp70 chaperone controls proteostasis and anti-stress responses in rapidly renewing cancer cells, making it an important target for therapeutic compounds. To date several Hsp70 inhibitors are presented with remarkable anticancer activity, however their clinical application is limited by the high toxicity towards normal cells. This study aimed to develop assays to search for the substances that reduce the chaperone activity of Hsp70 and diminish its protective function in cancer cells. On our mind the resulting compounds alone should be safe and function in combination with drugs widely employed in oncology. We constructed systems for the analysis of substrate-binding and refolding activity of Hsp70 and to validate the assays screened the substances representing most diverse groups of chemicals of InterBioScreen library. One of the inhibitors was AEAC, an N-amino-ethylamino derivative of colchicine, which toxicity was two-orders lower than that of parent compound. In contrast to colchicine, AEAC inhibited substrate-binding and refolding functions of Hsp70 chaperones. The results of a drug affinity responsive target stability assay, microscale thermophoresis and molecular docking show that AEAC binds Hsp70 with nanomolar affinity. AEAC was found to penetrate C6 rat glioblastoma and B16 mouse melanoma cells and reduce there the function of the Hsp70-mediated refolding system. Although the cytotoxic and growth inhibitory activities of AEAC were minimal, the compound was shown to increase the antitumor efficiency of doxorubicin in tumor cells of both types. When the tumors were grown in animals, AEAC administration in combination with doxorubicin exerted maximal therapeutic effect prolonging animal survival by 10–15 days and reducing tumor growth rate by 60%. To our knowledge, this is the first time that this approach to the high-throughput analysis of chaperone inhibitors has been applied, and it can be useful in the search for drug combinations that are effective in the treatment of highly resistant tumors.

## Introduction

Most of human tumors are known to contain high quantities of Hsp70 chaperone, suggesting that the protein is vital for the proper function of cancer cells^1^. Because of the cytoprotective power Hsp70 reduces the sensitivity of tumors to anti-cancer drugs (such as doxorubicin, etoposide, cisplatin, and others collectively known to induce apoptosis)^2^, an effective therapy should be at least partially based on targeting chaperone activity in cancer cells. Inhibiting such activity would result in an improved response to chemotherapy with less severe side effects.

Some of anti-chaperone substances can inhibit the efficacy of the heat-shock response by reducing the heat shock factor 1-mediated transcription of heat shock protein genes, similar to the mechanisms of compounds such as triptolide^3^ or KNK-437^4^. Another mechanism of reducing chaperone activity is to inhibit basing on Hsp70 folding mechanism by binding the protein or by affecting its interactions with co-chaperones, belonging to DNAJ or Bag-Hsp110 family of proteins^5^. One of the compounds recognizing ATP-binding domain on the Hsp70 molecule was MKT-077; it inactivated the chaperone function and inhibited the growth of human tumor cell lines, even when applied at low concentrations^6,7^. Another Hsp70 binder, 2-phenylethynesulfonamide (PES), dissociates Hsp70 and substrate proteins. Treatment of tumor cells with PES promoted protein aggregation and impaired autophagy, which led to the suppression of Myc-induced lymphomagenesis^8^. Similar anticancer effects were obtained by using VER-155008, which is an adenosine-derived inhibitor that targets the ATP-binding domain of Hsp70 (HSPA1A) and Hsc70 (HSPA8). Cells of several tumor lines, particularly breast carcinoma and colon cancer, treated with VER-155008 exhibited reduced growth rate and apoptosis^9^. Recently, five piperidine derivatives were discovered using the rational design approach as novel Hsp70 inhibitors. These compounds significantly inhibited the proliferation of cancer cells belonging to 16 different lineages^10^.

Promising results have been obtained with JG98, a novel compound that inhibits the interaction between Hsp70 and its Bag-3 co-chaperone. This substance blocked pro-survival signaling and inhibited proliferation in a variety of cancer cells^11^.

Design of Hsp70-binding compounds is currently performed using methods of molecular simulation and analysis of Hsp70 binding to substances, both known or newly developed affinity probes^12^. The common disadvantage of Hsp70 chaperone inhibitors is their high toxicity displayed in cell and animal cancer models; this property makes their application in oncological clinic questionable. Indeed, the search for the safer inhibitors that can serve as sensitizers of tumor is required and we chose such way of anti-Hsp70 drug design.

The aim of this study was to develop systems to search for substances that reduce the chaperone activity of Hsp70 and reduce its protective power. We used a method based on two key activities of the protein: (1) the ability to recognize and bind denatured protein substrates, and (2) to refold preliminarily denatured polypeptide, causing the recovery of its enzymatic activity. The application of these two assays to the analysis of a number of synthetic compounds resulted in the discovery of a negative regulator of Hsp70 activity that was found to sensitize melanoma and glioblastoma cells to the cytotoxic effect of doxorubicin.

## Results

### Screening of chemical library and a discovery of an active substance

**Fig. 1:**
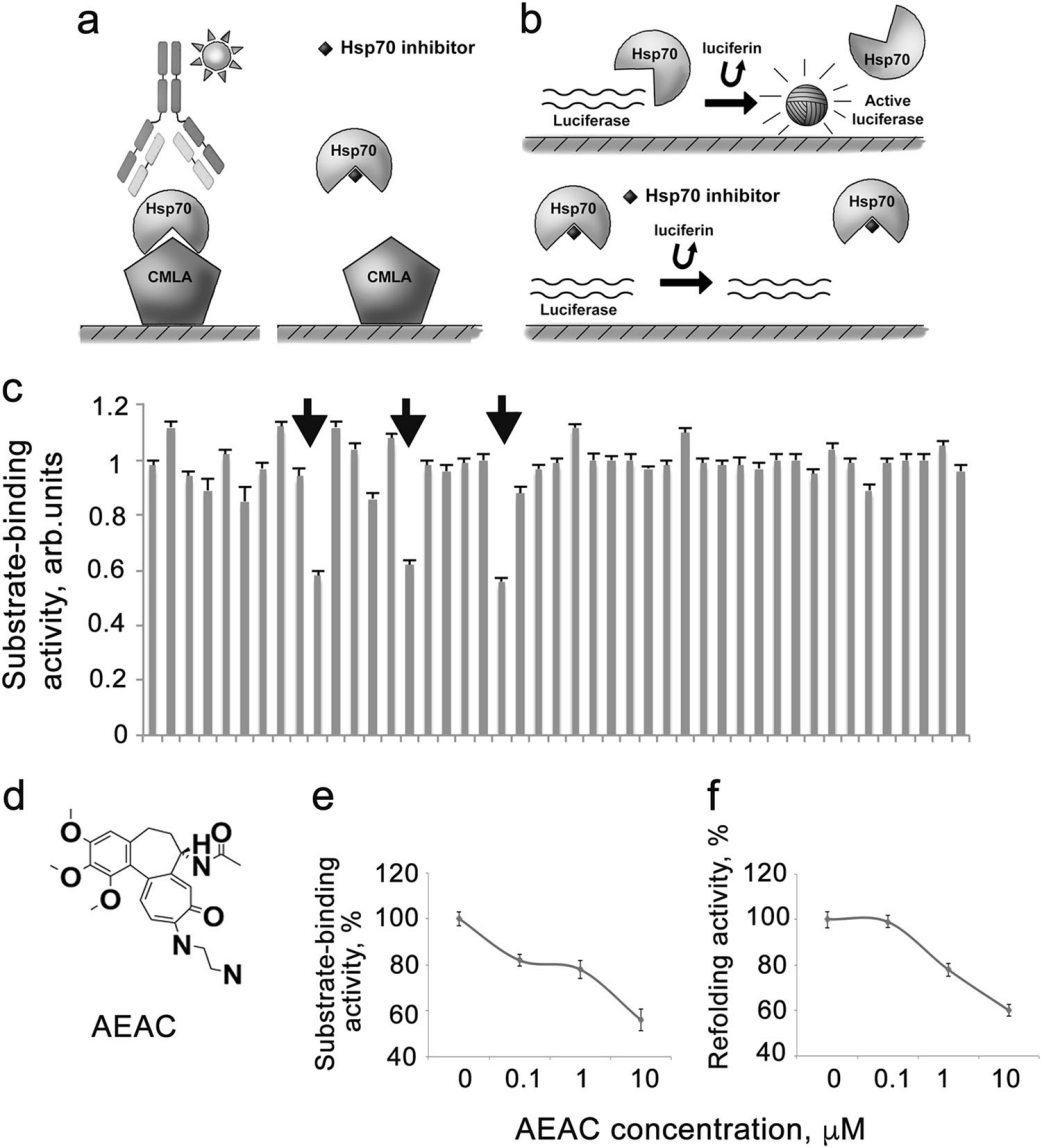
High-throughput screening identifies substances with the capacity to inhibit Hsp70 chaperone activity **a** Principle diagram of substrate-binding assay; **b** Principle diagram of refolding assay (see Materials and Methods section for detailed explanation); **c** Example of a readout of substrate-binding assay, which reveals three potential inhibitors of Hsp70 substrate-binding activity, pointed by arrows; **d** Formula of (S)-N-(10-((2-aminoethyl)amino)-1,2,3-trimethoxy-9-oxo-5,6,7,9-tetrahydrobenzo [a]heptalen-7-yl acetamide, AEAC; **e** AEAC inhibits substrate-binding activity and **f** refolding activity of purified Hsp70 in a dose-dependent manner

The first cycle of screening of potential Hsp70 inhibitors was performed using a substrate-binding assay in which the ability of a compound to reduce the Hsp70 binding to carboxymethylated lactalbumin was measured; see Fig. 1a for details. To validate the function of the substrate-binding assay, we used known modulators of Hsp70 and found that 1 μM MKT-077 and 1 μM VER-155008 reduced the binding of the chaperone to CMLA by 27 and 19%, respectively (Supplementary Figure 1).

Using the assay, we analyzed a group of 620 compounds and found approximately 30 substances that reduced the substrate-binding activity by 20–40% (Fig. 1c). After that we stopped the screening and focused on the further characterization of the hits. First, the inhibitors of substrate-binding activity underwent further analysis with the refolding assay. Using this test, we were able to measure the degree of denatured luciferase recovery when the enzyme was incubated with pure recombinant Hsp70 and with total protein extract of K-562 cells that contained a full set of co-chaperones.

AEAC, a synthetic derivative of colchicine, is one of compounds that we found to have Hsp70-inhibiting activity in both assays we used. Its chemical name is (S)-N-(10-((2-aminoethyl)amino)-1,2,3-trimethoxy-9-oxo-5,6,7,9-tetrahydrobenzo [a]heptalen-7-yl acetamide and the chemical formula is presented in Fig. 1d. AEAC was chosen for further analysis because its parental compound demonstrated therapeutic effects for several pathologies, including cancer. We estimated the concentration-dependence of the inhibitory capacity of AEAC in both test systems. The compound suppressed substrate-binding activity and luciferase re-activation at a concentration of 1 μM (Figs. 1e, f). We compared substrate-binding and refolding activity of AEAC and colchicine and found that these activities of colchicine were much lower (Supplementary Figures 2a and 2b). We concluded that colchicine did not demonstrate Hsp70 chaperone-modulating activity and omitted it from the further analysis.

### Hsp70 binds AEAC with high affinity

**Fig. 2:**
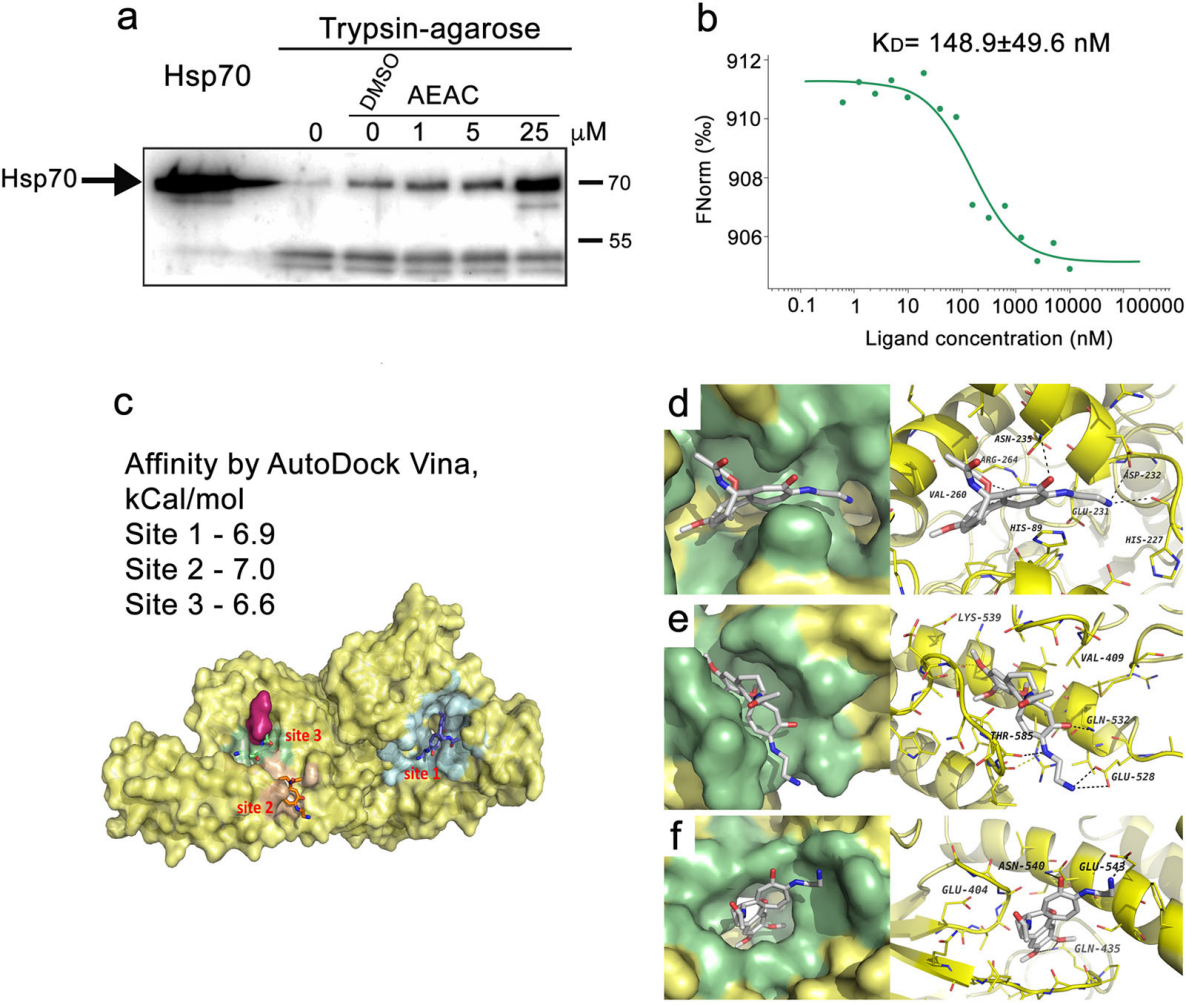
AEAC physically interacts with Hsp70 molecules **a** Data from DARTS assay. The products of Hsp70 trypsinolysis in the presence of AEAC in concentrations indicated were subjected to electrophoresis and immunoblotting using an anti-Hsp70 antibody; **b** Hsp70–AEAC binding measured by MTS. Unlabeled AEAC (20 μM–0.61 nM) was titrated into a fixed concentration of labeled Hsp70 (50 nM); **c** AEAC has three possible binding sites on the Hsp70 molecule. This figure represents the most probable positions of AEAC on the Hsp70 molecule **d-f**

The reduction of substrate-binding and refolding activity described previously led us to suggest that AEAC is able to physically bind the Hsp70 molecule (Fig. 1). To demonstrate this, we used a DARTS assay with modifications^13^. Pure recombinant Hsp70 was first incubated with trypsin-agarose beads in the presence of AEAC, which was used at different molar ratios to the Hsp70 protein. The complex was then analyzed using immunoblotting and the RS polyclonal antibody; this antibody recognizes the full molecule Hsp70 band and its proteolytic fragments (Fig. 2a). Trypsin caused the proteolysis of Hsp70 and, in the absence of AEAC, we observed the disappearance of the major 70 kDa band. The addition of AEAC to the incubation mixture prevented trypsin-mediated proteolysis in a dose-dependent manner, suggesting that the ligand binds Hsp70 in micromolar concentrations (Fig. 2a). Unexpectedly, dimethyl sulfoxide (DMSO) was also found to protect the Hsp70 molecule from trypsinolysis, although to a lesser extent compared with AEAC. Another assay used to estimate the affinity of the AEAC–Hsp70 complex was microscale thermophoresis. For these studies, we mixed pure Hsp70 with AEAC in different concentrations and obtained the typical curve showing the dependence of normalized fluorescence (∆Fnorm) on the concentration of the ligand, AEAC, which gave us the value *K*_*D*_ = 148.6 ± 49.6 nM (Fig. 2b).

To localize possible binding sites for AEAC on the Hsp70 molecule, we performed blind molecular docking of the ligand to the Hsp70 structure followed by verification using molecular dynamics simulations. Because the full molecule of human Hsp70 has not yet been crystallized, we performed docking to ATP-binding and substrate-binding domains separately. Three probable sites that demonstrated affinity values higher than 6.6 kCal/mol were found: site one (Fig. 2c) is located on the ATP-binding domain and sites two and three are located on the substrate-binding domain (SBD) (Fig. 2c). Site one is located on the protein surface opposite to the inter-subunit contact (Fig. 2d). Site two is positioned between the SBD-α and SBD-β regions of Hsp70 (Fig. 2e), which is in the pocket near the helical part of the SBD. Site three is located within the substrate-binding channel (Fig. 2f).

### AEAC penetrates inside living cells and inhibits chaperone activity of Hsp70

**Fig. 3:**
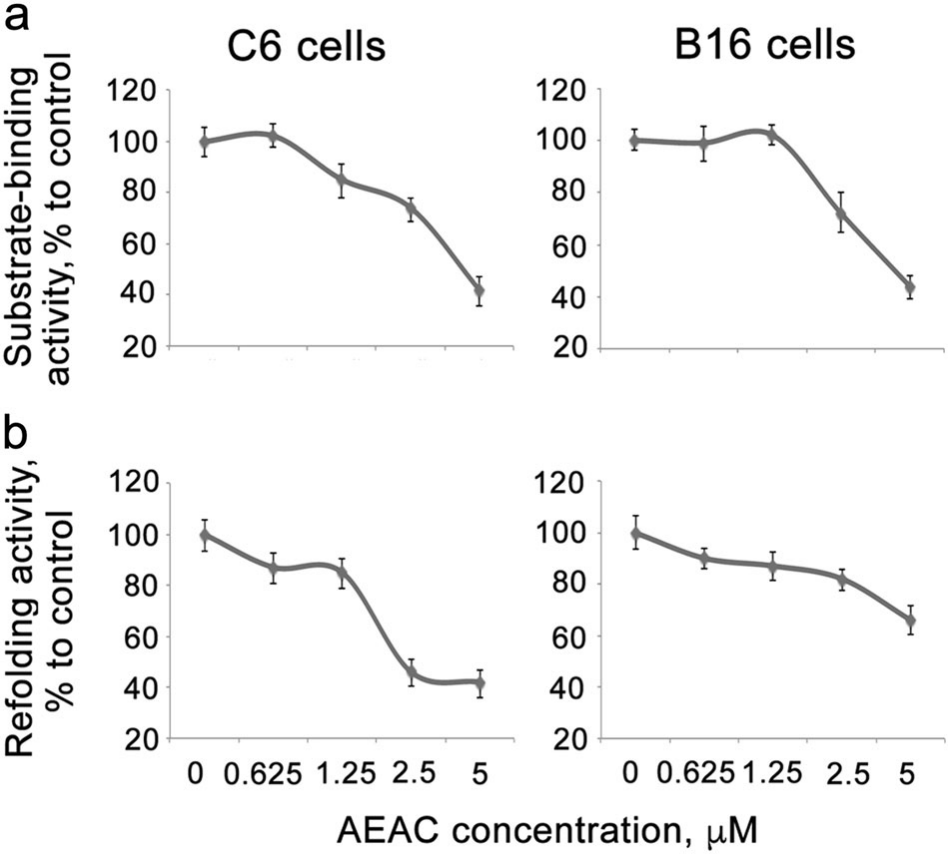
AEAC is able to penetrate inside tumor cells and to inhibit chaperone ability of intracellular Hsp70 **a** Rat glioma C6 cells (left) and mouse melanoma B16 (right) cells were heat-shocked in the presence of AEAC in concentrations indicated. After 6 h of recovery, cells were lysed and cell lysates were used for the substrate-binding assay; **b** C6 and B16 cells were transiently were transfected with a pcDNA3 plasmid contained luciferase gene, heat shocked in the presence of AEAC, and cell lysates were used for the luciferase-refolding assay

To investigate whether AEAC is able to penetrate inside living cells and to affect chaperone activity of intracellular Hsp70, we measured its capacity to decrease substrate-binding and refolding activity in cells subjected to a mild heat shock. C6 rat glioblastoma and B16 mouse melanoma cells were heated at 43 °C for 30 min to increase the intracellular Hsp70 content, and were then incubated with AEAC (Fig. 3a). Freshly prepared cell lysates (see the Materials and Methods section for details) were transferred to the wells of the 96-well microplate with immobilized CMLA. After incubation and washing, RS antibody was added, as described in the Materials and Methods. Incubation with 2.5 μM AEAC was found to reduce the binding capacity of Hsp70 (from C6 cell lysate) to CMLA by 50% as compared to non-treated C6 cells. This finding provides evidence that the compound can regulate Hsp70 activity inside a cell (Fig. 3a). The same inhibitory effect was observed for 2.5 μM AEAC in heat-stressed B16 melanoma cells in which the substrate-binding capacity of endogenous Hsp70 was lowered by 30% in cells that were incubated with AEAC (Fig. 3a).

To understand whether AEAC can also inhibit the refolding activity of intracellular chaperone, we transfected C6 and B16 tumor cells with a plasmid-containing luciferase gene using a cytomegalovirus promotor. Heat shock at 43 °C for 30 min caused an almost 100-fold reduction of enzyme activity in both C6 and B16 cells; the luminescence recovered spontaneously within 6 h at 37 °C, probably due to the accumulation of Hsp70 in cells after heat shock (data not shown). The addition of AEAC prior to heat stress caused a decline in re-activation dynamics, which suggested that the compound not only penetrated cells of both types, but also damaged Hsp70-based chaperone machinery (Fig. 3b). In both cell lines the reduction of refolding activity was most effective with 2.5 μM AEAC. There was no inhibition of luciferase recovery in control cells that were incubated only with the vehicle, DMSO.

### The anticancer effects of combining AEAC and doxorubicin

**Fig. 4:**
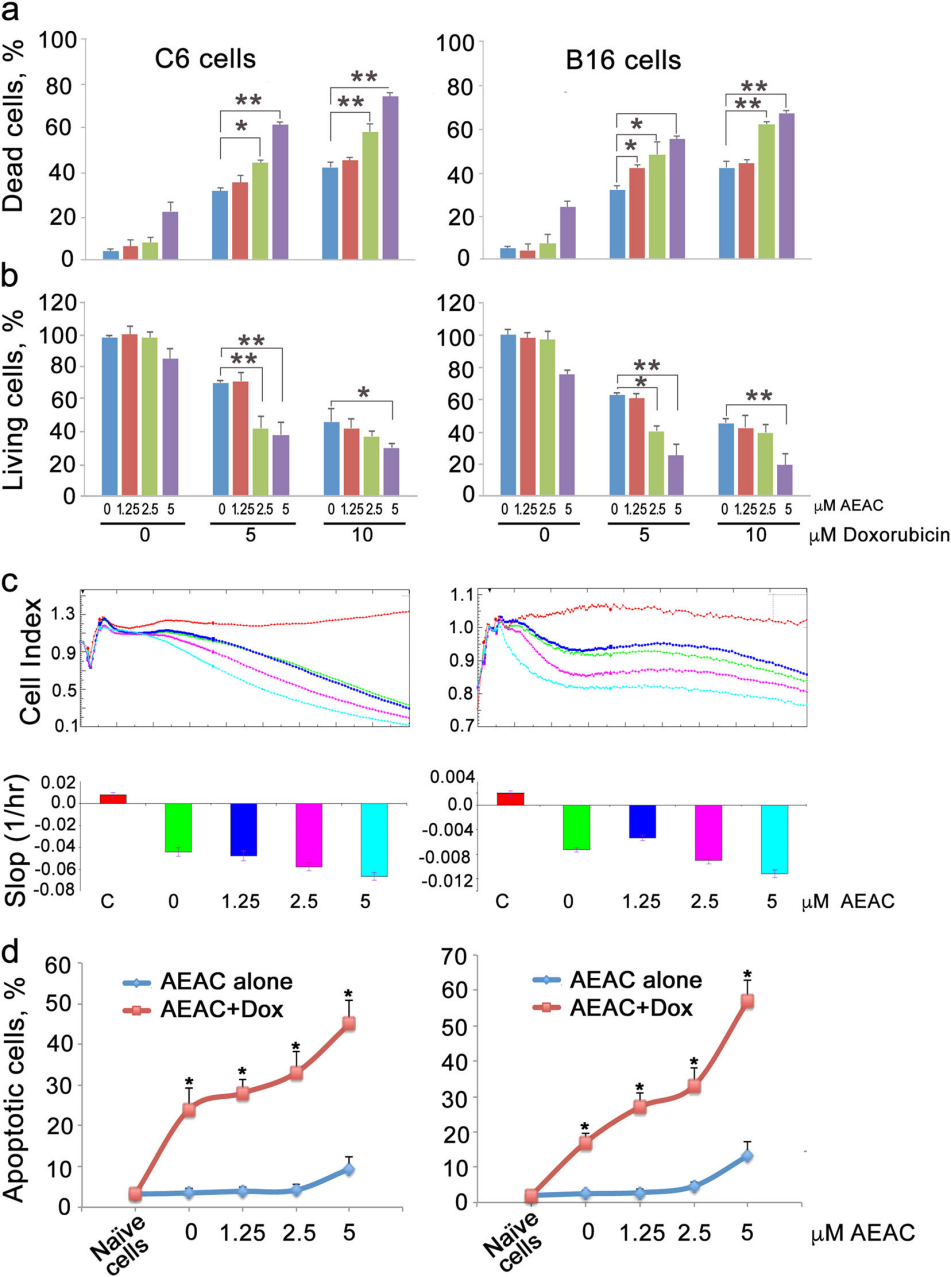
AEAC increases the effects of doxorubicin on the viability of C6 glioma and B16 melanoma cells C6 glioma cells (left panel) and B16 melanoma cells (right panel) were seeded in the wells of 96-well plates and incubated with AEAC alone or in combination with doxorubicin for 24 h. Cell media from the wells was then used to measure LDH activity using the Cytotox96 kit **a** and the cells in the wells were used for the MTT assay **b** (each single concentration of drugs, or combination of drug, was used in triplicates). Data is presented as the means ± standard error of the mean (SEM). A statistical difference was determined by a value of *p* < 0.05; **c** Proliferation rates of C6 or B16 cells incubated with AEAC and doxorubicin alone or in combination. The data are shown as cell index graphs. **d** Cells were incubated with AEAC alone or in combination with doxorubicin, and cells with apoptotic nuclei were calculated. All experiments were made in triplicates. Statistical significance is indicated as * *p* < 0,0.1

Because AEAC has been shown to penetrate living cells, interact with Hsp70, and inhibit Hsp70 substrate-binding and refolding activities (and, therefore, to impair protective function of the chaperone), we suggest that the compound could reduce resistance of tumor cells to anti-cancer drugs. First, we analyzed the effects of AEAC alone using a CytoTox96 kit and an MTT assay. According to the data of CytoTox96 assay, the IC_50_ value was 194.55 μM for C6 cells and 97.85 μM for B16 cells. The toxicity of AEAC in concentrations of 1.25 μM and 2.5 μM was insignificant and comprised 7.6 ± 2.9 and 8.8 ± 1.8, respectively, for C6 cells and 3.8 ± 3.4 and 8.7 ± 4.0, for B16 cells (Fig. 4a). Similar results were obtained with the aid of the MTT test; 1.25 and 2.5 μM AEAC did not affect cell viability, whereas application of 5 μM AEAC led to a reduction in viability to 25.6 ± 3.4% for C6 cells and 24.1 ± 1.8% for B16 cells (Fig. 4b).

To test the effect of AEAC in combination with an anticancer drug, we used doxorubicin, which is currently used in clinical oncology. Combinations consisted of doxorubicin in two concentrations (5 and 10 μM) and AEAC in concentrations of 0, 1.25, 2.5 and 5 μM. The data from measuring LDH activity in medium (CytoTox96 assay) showed that the addition of AEAC to cell culture increased the toxicity of doxorubicin in both concentrations. 2.5 μM AEAC increased the toxicity of doxorubicin (5 μM) against C6 cells from 32.1 ± 0.7% to 47.6 ± 0.8% (Fig. 4a). Similarly, 1.25 μM AEAC increased the toxicity of doxorubicin (5 μM) from 32.0 ± 0.7% to 41.5 ± 1.1% in B16 cells (in comparison, the toxicity value for 10 μM doxorubicin alone was 40.9 ± 3.9%) (Fig. 4a, right panel). We confirmed these data using an MTT test and demonstrated that in the presence of 2.5 μM of AEAC, 5 μM doxorubicin inhibited viability of C6 cells by 39% and of B16 cells by 35%. The viability in the presence of 10 μM of doxorubicin in C6 cells was 50.9 ± 1.8% and in B16 cells 45.9 ± 2.0% (Fig. 4b).

To verify the data obtained from the CytoTox96 and the MTT assays, we used xCELLigence technology, which allowed us to measure cell index in real time. The parameter is based on impedance measurements and reflects the number of cells attached to the surface electrode of a multi-well chamber. We analyzed the dynamics of cell death in the cultures of C6 and B16 cells in the presence of 5 μM doxorubicin in combination with AEAC in concentrations of 0, 1.25, 2.5 and 5 μM and found that AEAC increased the toxicity of doxorubicin in a dose-dependent manner in both C6 and B16 cell populations (Fig. 4c).

Because doxorubicin is a potent inducer of apoptosis^14^, we analyzed whether AEAC can increase the pro-apoptotic effects of the drug. The percentage of cells that demonstrated apoptotic nuclear morphology (visualized by Acridine orange staining) increased from 25.2 ± 2.9% in cells incubated with doxorubicin alone to 33.4 ± 4.0% when doxorubicin was combined with 2.5 μM AEAC in C6 cells and from 22.8 ± 1.6% to 32.4 ± 2.5% in B16 cells. Importantly, AEAC alone in concentration 2.5 μM caused apoptosis in less than 5.5% of C6 and B16 cell populations (Fig. 4d).

### Combined effects of anticancer drugs with AEAC in vivo

**Fig. 5:**
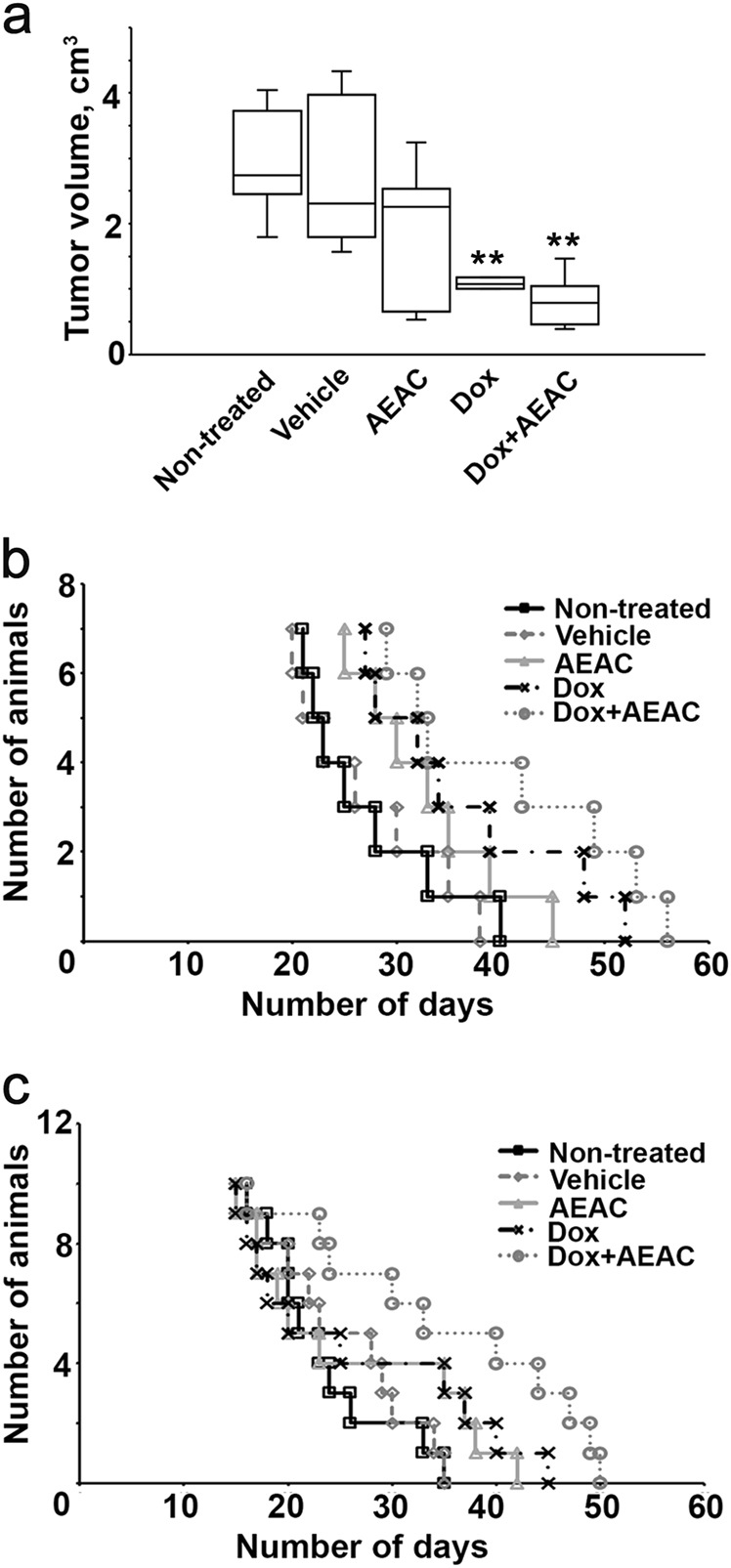
AEAC enhances the effect of doxorubicin on suppressing tumor growth, which leads to an increase in the survival of tumor-bearing animals **a** Tumor volume and **b** life span of tumor-bearing mice were estimated. C57/Bl6 mice were inoculated subcutaneously with 10^6^ B16 cells. Seven days later, doxorubicin or AEAC alone or in combination in DMSO was applied every two days to the injection area; **c** C6 glioma cells were grafted intracranially, and seven days later rats were injected intraperitoneally with doxorubicin or AEAC alone or in combination. The therapy continued to day 27 after grafting, and application was repeated each second day. The cumulative proportion surviving Kaplan–Meier curve is presented. Statistical significance is indicated as ** *p* < 0,0.1

To determine whether AEAC is able to elevate antitumor capacity of doxorubicin, we used two animal models: mouse melanoma B16 growing subcutaneously and the intracranial rat C6 glioblastoma^15–17^. After tumor cell inoculation and treatment with doxorubicin alone or in combination with AEAC (see Materials and Methods for details), we measured tumor size in B16-bearing mice on the day 20 after tumor grafting. Treatment with doxorubicin alone inhibited tumor growth by approximately 58.22%; the average tumor size was 1.25 ± 0.59 cm^3^ vs 2.98 ± 0.82 cm^3^ in non-treated group or 2.80 ± 1.08 cm^3^ in the group treated with the vehicle. Combined action of doxorubicin with AEAC slowed tumor growth even more; the average tumor size in this group was 0.80 ± 0.31 cm^3^ (Fig. 5a). Interestingly, treatment with AEAC alone also suppressed tumor growth by 36.95%; the average tumor size in this group was 1.88 ± 1.05 cm^3^ (Fig. 5a). The representative photos of mice from different groups on the 20th day after B16 cells injection are presented on the Supplementary Figure S3.

We also evaluated the lifespan of tumor-bearing mice after various treatment modes. Treatment with doxorubicin alone increased the lifespan by 10–12 days, compared to the control group (37.1 ± 3.6 days vs. 27.4 ± 2.6 days). Combined therapy with doxorubicin and AEAC prolonged survival of tumor bearing mice for an average of 5 additional days (42 ± 4.1 days) compared to mice treated with doxorubicin only. The therapy with AEAC alone also was effective, and it resulted in a 5-day delay in animal death compared to control groups (Fig. 5b).

A similar animal survival profile was demonstrated in rats with intracranial C6 glioblastoma. The combined doxorubicin and AEAC therapy was effective and the lifespan was prolonged by 12 ± 2.2 days compared to the non-treated group. Interestingly, the therapeutic efficacy of doxorubicin alone and AEAC alone was much lower; the death was postponed for 3.2 ± 1.9 days in the group treated with doxorubicin and for 2.7 ± 1.7 days in the AEAC group compared to the control group (Fig. 5c).

## Discussion

The fact that Hsp70 is found in a high quantity in numerous tumors is well documented and prompts researchers to search for a way to inhibit the chaperone. To date, over 20 small-molecule inhibitors of Hsp70 have been identified and most of them are shown to possess anti-cancer activity in a variety of cell and animal models. Hsp70 inhibitors are created using rational drug designs to identify a specific part of the chaperone molecule that can serve as the target for the careful selection of the most affine one^18^. It is clear that scaffold compounds that are generated in these experiments do not necessarily inhibit chaperone activity or protective function. To specifically target the chaperone capacity of Hsp70, we modified two assays that have been previously used for the same purpose^19,20^, and adapted them for high-throughput analysis of chemical compounds.

The substrate-binding assay revealed approximately 30 modulators of Hsp70 activity from the selection of more than 600 representatives of maximally diverse chemicals collected in InterBioScreen library (Fig. 1). The hits were investigated further using a refolding assay that resulted in 10 to 12 (of the 30 previously chosen) Hsp70 inhibitor compounds retaining both the substrate-binding and refolding activities. The absence of correlation between the efficacy of denatured protein recognition and refolding activity found for remaining 20 compounds led us to suggest that the recognition of the substrate and the conversion of its molecule to a native state may be differently regulated. An example of similar discoordination between the client protein binding and ATP-ase was demonstrated for a bacterial member of the Hsp70 family ^21^. Notably, we have not employed ATP in substrate-binding assay because its addition to reaction mixture completely inhibited the Hsp70-substrate interaction by passing the chaperone to intermediate state. This was true irrespective of whether the interaction occurred with a substrate (CMLA) and pure Hsp70 or the chaperone existing in cell extract as shown earlier^19^.

To confirm the feasibility of the assays used in the study, we chose MKT-077 and VER-155008, which bound different domains of Hsp70^18^, and found that the substances in 1 μM concentration inhibited luciferase recovery by 18 and 14%, respectively (Supplementary Figure 1). Indeed, both compounds inhibited substrate-binding activity of Hsp70.

To expand the application of both assays to the simultaneous analysis of penetrative activity of the compounds and their ability to regulate Hsp70 chaperone function we employed tests in vivo imitating both above assays. The system comprising cells expressing luciferase is similar to that of Nollen with coauthors and allows to study the recovery of luciferase under the action of Hsp70-Hsp40 chaperones in cultured cells^22^. We employed this system and found that AEAC not only penetrated cells and proved its relevance for the screening of Hsp70 inhibitors. Another test we developed was substrate-binding assay transmitted to in vitro format. Using the test, we proved that AEAC not only penetrated cells of completely different origin and also dose-dependently reduced the substrate-recognition activity of Hsp70. We conclude that the assays developed in this study can be applied to the screening of a wide spectrum of Hsp70 modulators, and that they are less time-consuming and labor-intensive than the current methods.

Since chaperone-inhibitory activity of AEAC may mean that its molecule binds Hsp70, we investigated this suggestion using three different methods and found that according to data of microscale thermophoresis the *K*_*D*_ value is approximately 0.15 μM. Similar values of the constant have been reported for other small-molecule binders of Hsp70, which suggests that the efficiency of their anti-Hsp70 effects is similar^23^. Using molecular docking and dynamics simulation we found that there are three binding sites for AEAC on Hsp70 molecule (Figs. 2c,d). It is meaningful that site two locates near the helical part of the SBD that is involved in the conversion from open to closed conformations of Hsp70 (Fig. 2e). Recently, this site was found to be potentially druggable on DnaK^24,25^.

One of the goals of the study was to explore the possibility of using AEAC in therapeutic protocols in combination with doxorubicin, a well-established drug. We first analyzed the effects of the compound alone and found that AEAC is toxic at concentrations that exceed 5 μM, and values of IC_50_ obtained for C6 and B16, 195 and 98 μM, respectively, are higher than for the most other Hsp70 modulators. For example, MKT-077, PES-Cl, and VER-155008 are toxic to human melanoma A375 and adenocarcinoma H1299 cells, with IC_50_ in the micromolar range^26^. In a more recent study, Zeng *et al*. analyzed the activity of 67 novel piperidine derivatives in 16 drug-resistant cancer cells and demonstrated that five successful compounds have an IC_50_ of approximately 1 μM^10^. We conclude that AEAC alone does not affect cell viability and growth as strongly as other Hsp70 inhibitors do, and tested it in combination with doxorubicin for anti-tumor activity in rat glioblastoma C6 and mouse melanoma B16 cells.

The results show that a concentration of 2.5 μM AEAC enhances the cytotoxic effect of 5 μM doxorubicin up to values corresponding to 10 μM doxorubicin, e.g., AEAC demonstrated its ability to strongly increase the sensitivity of cancer cells to a classic antitumor drug (Fig. 4). The same phenomenon of AEAC-mediated sensitization of tumors was observed in experiments in vivo with tumors of quite diverse origin; in both cases we observed the prolongation of survival of animals with melanoma or glioma by 53.3 and 50.8% that was a better result than after treatment with doxorubicin alone (Fig. 5).

We found only a few reports concerning application of anti-chaperone compounds in combination with antitumor drugs. First, the combinations of triptolide (a known inhibitor of the heat shock response) with several anticancer drugs were shown to be extremely effective against human breast cancer cells grafted onto nude mice^27^. Despite the promising effects of the combination, this study does not include detailed information about the toxicity of triptolide alone, which is a limiting factor in the clinical application of the drug. Secondly, in a study that is similar to our present work, McKeon et al^28^. used a combination of pifithrin-μ (PES) and cisplatin or oxaliplatin and found strong synergistic effects in cultures of PC-3 prostate cancer cells and HT-29 colorectal cancer cells. It is clear that for the development of more effective therapeutic tools based on well-established anticancer drugs in combinations with Hsp70 chaperone inhibitors the search for concrete antitumor partners is needed.

In conclusion, our work has demonstrated a few novel test systems that support the search of possible modulators of Hsp70 chaperone proteins. One such molecule was identified as a potent co-factor in combinational anticancer therapy.

## Materials and methods

### Compounds

The library of InterBioScreen (http://www.ibscreen.com) was employed to screen for the inhibitors of Hsp70 activity. The compounds chosen for the screen (620 pcs) had chemical structures belonging to maximally diverse chemical groups. The chemicals were dissolved in DMSO and stored at −20 °C.

### Purification of Hsp70 and measurement of its chaperone activity

Recombinant human Hsp70 was purified from bacteria that was transformed with a pMSHsp70 plasmid using a two-step chromatography procedure previously described in^29^. The substrate-binding activity of Hsp70 was measured using a modified enzyme immunoassay^19,30^. Human recombinant Hsp70 (100 ng/ml) was mixed with different inhibitors in a buffer that contained 20 mM Tris HCl, 20 mM NaCl, and 10 mM MgCl_2_ (pH 7.5), and then transferred to the wells. Anti-Hsp70 polyclonal antibody (RS) was applied^15^, followed by incubation with anti-rabbit IgG conjugated with peroxidase (Jackson Immunochemicals, USA). After the addition of tetramethylbenzene in a citrate buffer (pH 4.5) containing also hydrogen peroxide, the intensity of the staining was measured using a Charity Fluorofot multipurpose reader (Probanauchpribor, Russia).

The luciferase refolding assay was designed according to the protocol reported by Cassel et al.^20^, with some modifications. Briefly, pure luciferase was inactivated by incubation in the buffer contained Hepes-KOH^16^ pH 7.5, 10 mM KCl, 5 mM MgCl_2_, and 8 M urea at room temperature for 30 min^16^. The treatment caused the loss of almost 99% of activity. The luciferase solution was diluted 100-fold and mixed with an equal volume of refolding mixture that contained heat shocked K-562 cell lysate (final concentration equal to 500 μg/ml), which served as the source of the co-chaperones, 60 μg/ml recombinant Hsp70, 10 mM creatine phosphate, 16 U/ml creatine phosphokinase, 2 mM ATP, and 2 mM DTT. The mixture was transferred to a 96-well plate (150 μl in each well) and analyzed compounds were added to wells in various concentrations. The microplate was shaken at 37 °C for 30 min. Next, 20 μl of Bright-GLO Luciferase Assay System Buffer (Promega, USA) was added to an equal volume of refolding mixture. Luciferase activity was measured by luminometric analysis, which was completed using the Charity Fluorofot multipurpose reader (Probanauchpribor, Russia) and expressed as percentage of the activity before inactivation.

### Analysis of interaction between Hsp70 and AEAC

The examination of binding of small molecules to Hsp70 was performed using molecular docking, drug affinity responsive target stability (DARTS) and microscale thermophoresis, see Supplementary materials.

### Cells

C6 rat glioblastoma and K562 human erythroleukemia cells were obtained from the Russian Collection of Cell Cultures (Institute of Cytology, Russian Academy of Sciences, St. Petersburg). C6 cells were grown in DMEM-F/12 medium (Biolot, Russia); K562 cells were grown in Roswell Park Memorial Institute medium (Biolot, Russia). B16-F10 mouse melanoma cells were obtained from Dr. L. Sistonen (Turku Biocenter, Finland) and were maintained in Dulbecco’s modified eagle medium (DMEM) (Biolot, Russia), 10% fetal calf serum (Paneco, Russia), penicillin G (100 ME/ml), and streptomycin (100 μg/ml) (Biolot, Russia). Cells were incubated in a humidified incubator at 37 ^o^C with 6% CO_2_.

### Measurements of AEAC effects on intracellular Hsp70 activity

#### Substrate-binding assay

The effects of AEAC on intracellular substrate-binding Hsp70 activity were explored, as described by^19^. Briefly, CMLA (10 μg/ml in phosphate-buffered saline, PBS) was applied on the surface of a 96-well microplate. Non-specific binding was then eliminated by applying 5 mg/ml bovine serum albumin in PBS. The extracts were obtained from C6 or B16 cells that were incubated with AEAC for 30 min and then subjected to heat shock at 43 °C for 30 min to induce Hsp70 accumulation. Thirty minutes after heat shock, the cells were lysed by freeze-thaw cycles in 20 mM Tris-HCl (pH 7.5), 50 mM NaCl, 0.5% Triton × 100, 2 mM ethylenediaminetetraacetic acid, and 1 mM phenylmethlsulfonyl fluoride. The lysate of C6 or B16 cells or pure Hsp70 in buffer (20 mM Tris-HCl, 20 mM NaCl, and 10 mM MgCl_2_ (pH 7.5) were mixed with an inhibitor and applied to wells. Next, RS polyclonal antibody was added followed by anti-rabbit IgG conjugated with peroxidase (Jackson Immunochemicals, USA). After the addition of tetramethylbenzene in a citrate buffer (pH 4.5) with hydrogen peroxide, the degree of staining was measured using a Charity Fluorofot multipurpose reader (Probanauchpribor, Russia).

#### Refolding assay

For the in vitro refolding analysis, which provided additional support to the suggestion of AEAC permeability, C6 or B16 cells were transfected with a pcDNA3 plasmid that contained luciferase gene (kindly provided by Dr. D. Tentler, Institute of Cytology of RAS, Russia). Transfected cells were treated with AEAC and subjected to heat shock at C^o^C for 30 min to denature the enzyme. Eight hours later the cells were lysed in a solution containing 20 mM Tris-HCl, 150 mM NaCl, and 0.1% Triton X-100. Cell lysate from each sample (20 μl) was mixed with an equal volume of Bright-GLO solution, as described above.

#### Estimation of cell viability and apoptosis

To measure the amount of dead or growing cells, we used CytoTox96 kit (Promega, UK) and 3-(4,5-dimethylthiazol- 2-yl)-2,5-diphenyltetrazolium bromide (MTT) reagent (Sigma Aldrich, USA). C6 or B16 cells were placed in 96-well plates and after overnight cultivation AEAC was added in the concentration indicated. Twenty-four hours later, cytotoxicity and the viability value were measured. Acridine orange staining was used to measure the number of apoptotic cells. The xCelligence RTCA DP instrument (ACEA Biosciences®, Inc, USA) was used to measure the growth dynamics of C6 or B16 cells that were exposed to doxorubicin and AEAC.

#### Effects of Hsp70 inhibitors in combination with doxorubicin in vivo

C57Bl/6 female mice and male Wistar rats were purchased from an animal nursery (Rappolovo, St.Petersburg, Russia). Mice were subcutaneously grafted with 10^6^ B16 cells and divided into five groups, with seven mice in each group. Beginning on day seven after tumor cell inoculation, the groups were treated in different ways — group one was untreated (non-treated), group two (vehicle) was treated with DMSO alone, group three (AEAC) was treated with 2 mg/kg AEAC, group four (Dox) was treated with 1 mg/kg doxorubicin (Sigma-Aldrich, USA), and group five (Dox + AEAC) was treated with a combination of 2 mg/kg AEAC and 1 mg/kg Doxorubicin. AEAC was applied to a shaven area of skin surrounding the tumor, as described earlier^16^, and the application was repeated every second day until day 27 of the experiment.

Tumor length (L) and width (W) were measured using a sliding caliper. Measurements were taken for all groups on day 20 following inoculation with B16 cells. Tumor volumes were calculated using the formula (L × W^2^)/2.

Wistar rats were anesthetized before mounting in a stereotactic frame (David Kopf Instruments, Tujunda, CA), as described earlier^31^. Rats were divided into the same groups as described above, but 10 rats were included in each group. Treatment was started on day 7 after surgery. AEAC and doxorubicine were administered intraperitoneally and treatment continued to day 27 (one application every two days).

All animal experiments were carried out in accordance with the guidelines for the welfare of animals of the Institute of Cytology, Russian Academy of Sciences.

### Statistical analysis

Statistical analysis was performed using a one-way ANOVA test supplemented with the posthoc test. The results were considered to be statistically significant at *p* < 0.05 (*). All data presented represent a mean of at least three experiments. Standard deviation was plotted as error bars.

## Electronic supplementary material

Supplemental material
