# Supplementary material for: Sensitizing tumor cells to conventional drugs: HSP70 chaperone inhibitors, their selection and application in cancer models
Source: Cell Death Dis. 2018 Jan 18;9(2):41. doi: 10.1038/s41419-017-0160-y (PMC5833849; doi:10.1038/s41419-017-0160-y)
Supplement: Supplementary file 1 — Supplemental material [file 41419_2017_160_MOESM1_ESM.pdf]

SUPPLEMENTARY INFORMATION

"Sensitizing tumor cells to conventional drugs: HSP70 chaperone inhibitors, their selection and application in cancer models"

Vladimir F. Lazarev, Dmitry Sverchinsky, Elena R. Mikaylova, Pavel I. Semenyuk, Elena Y.Komarova, Sergey A. Niskanen, Alina D Nikotina, Anton V. Burakov , Viktor G. Kartsev, Irina V.Guzhova, Boris A.Margulis

MATERALS AND METODS

Analysis of interaction between Hsp70 and AEAC

Molecular docking

The search for a potential binding site on the Hsp70 molecule was performed using an AutoDock Vina software to do blind docking analysis (1). We used two target structures in humans: 1) Protein Data Bank (PDB) ID 3atv for ATP-binding domain, and 2) PDB ID 4po2 for the substrate-binding domain. In each case, only one chain was used for the docking, and the substrate molecule was ignored. The twenty best poses for each domain were selected for further analyses. Poses within the same pocket were grouped. Only one unique pose in every group was used for further analysis (in some cases there were two or three different poses in the one pocket). The poses around the C-end of the N-terminal domain and N-end of the C-terminal domain were rejected.

For the selected poses we performed molecular dynamics simulations using GROMACS 5.1 software (2). The ligand topology was created using the PRODRG server (3). The GROMOS 54a7 force field was used. For each pose we constructed an orthogonal simulation box with a distance of 2 nm from the border to the protein. Low-molecular ions (Na+) for the protein charge compensation were added and short simulations for pre-equilibration were performed. The duration of the main simulations was 100 ns and the step was 0.002 ps. The temperature was 300

K, and was measured using a v-rescale thermostat. Pressure coupling was performed using a Berendsen algorithm.

The trajectories obtained were analyzed to reject unstable poses. We rejected poses if the ligand left the pocket or if their fluctuation around the large pocket was big. For the stable poses, we determined the ligand-binding site by selecting the protein residues that had a distance from the ligand of less than 0.35 nm during the last 10 ns.

The molecular dynamics simulations were performed at the Supercomputing Center of Lomonosov at Moscow State University (4). For a clear visualization of the location of the predicted binding site, we roughly reconstructed the structure of the full Hsp70 molecule. For this we used the structure of the DnaK protein, which is a bacterial Hsp70 homolog. The structures of substrate-binding and ATP-binding domains were aligned to the corresponding domains of the DnaK structure (PDB ID 4jn4). The picture was made with the use of PyMOL software.

Drug affinity responsive target stability

For the drug affinity responsive target stability (DARTS) assay we used the protocol of Lomenick et al. (5), with some modifications described by Lazarev et al. (6); instead of the pure protease, we used trypsin immobilized on agarose (Sigma-Aldrich, USA). Before proteolysis, purified Hsp70 (1 µg in 20 µl) was incubated with binders at 4°C for 1 hr. Next, 2 µl of the trypsin–agarose was added to each sample and the mixture was incubated at 37°C for 45 min. The gel was separated by centrifugation, and the supernatant was analyzed by polyacrylamide gel electrophoresis and immunoblotting using RS anti-Hsp70 antibodies (7) and then anti-rabbit IgG conjugated with peroxidase (Jackson Immunochemicals, USA).

Microscale Thermophoresis (MST)

We used NanoTemper technology (8) to measure the binding capacity of (S)-N-(10-((2-aminoethyl)amino)-1,2,3-trimethoxy-9-oxo-5,6,7,9-tetrahydrobenzo [a]heptalen-7-yl acetamide (AEAC) to Hsp70. The Hsp70 was labeled with the dye NT-647 through N-hydroxysuccinimide (NHS) coupling, according to the manufacturer's protocol. In a typical microscale thermophoresis (MST) experiment, the concentration of NT-647-labeled Hsp70 was kept constant while the concentration of non-labeled AEAC was varied between 20 µM and 0.61 nM in MST buffer (20 mM HEPES buffer, pH 8.0, 200 mM NaCl, 1mM β-Mercaptoethanol, 0.05% Tween 20). After the 1hr incubation at room temperature, samples were loaded into MST NT. 115 premium glass capillaries and MST analysis was performed using the Monolith NT.115

(Nanotemper technology, Germany). The laser power was 20%. Data analysis was performed using Nanotemper Analysis software v.1.2.101.

REFERENCES

1. Trott O, Olson AJ. AutoDock Vina: Improving the speed and accuracy of docking with a new scoring function, efficient optimization, and multithreading. J Comput Chem 2010; 31:455–61

2. Pronk S, Páll S, Schulz R, Larsson P, Bjelkmar P, Apostolov R et al. GROMACS 4.5: a high-throughput and highly parallel open source molecular simulation toolkit. Bioinforma Oxf Engl 2013; 29:845–54

3. Schüttelkopf AW, van Aalten DM. PRODRG: a tool for high-throughput crystallography of protein–ligand complexes. Acta Crystallogr D Biol Crystallogr 2004; 60:1355–63

4. Sadovnichy V, Tikhonravov A, Opanasenko V, Voevodin V. “Lomonosov”: Supercomputing at Moscow State University. In Contemporary High Performance Computing: From Petascale toward Exascale Chapman and Hall/CRC, 2013; pp. 283–307. Jeffrey S. Vetter

5. Lomenick B, Hao R, Jonai N, Chin RM, Aghajan M, Warburton S et al.Target identification using drug affinity responsive target stability (DARTS). Proc Natl Acad Sci U S A 2009; 106:21984-9

6. Lazarev VF, Benken KA, Semenyuk PI, Sarantseva SV, Bolshakova OI, Mikhaylova ER et al. GAPDH binders as potential drugs for the therapy of polyglutamine diseases: design of a new screening assay. FEBS Lett 2015; 589:581-7

7. Shevtsov MA, Komarova EY, Meshalkina DA, Bychkova NV, Aksenov ND, Abkin SV et al. Exogenously delivered heat shock protein 70 displaces its endogenous analogue and sensitizes cancer cells to lymphocytes-mediated cytotoxicity. Oncotarget 2014; 5:3101–14

8. Jerabek-Willemsen M, Wienken CJ, Braun D, Baaske P, Duhr S. Molecular interaction studies using microscale thermophoresis. Assay Drug Dev Technol 2011; 9:342-53

Supplementary Figure S1

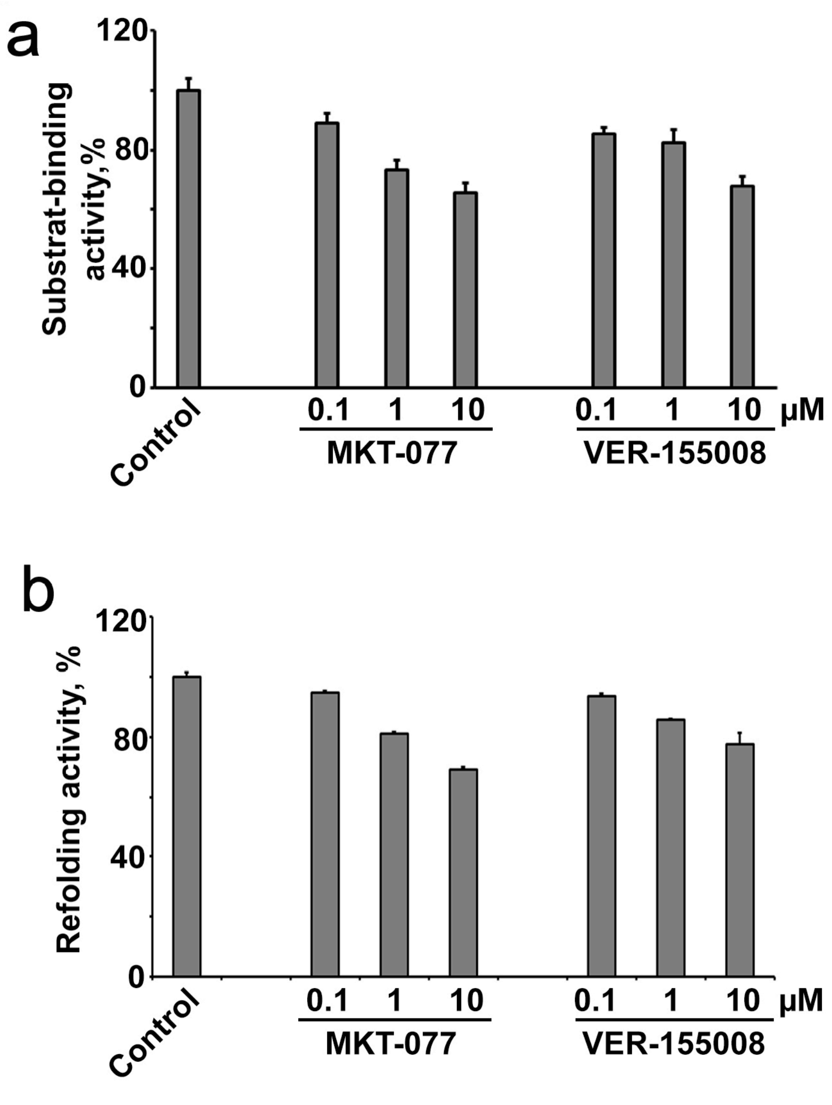

Supplementary Figure S1. Approbation of test systems for screening drugs that suppress the substrate-binding and refolding activity of Hsp70, using known chaperone inhibitors MKT-077 and VER-155008.

(A) Substrate-binding and (B) refolding assays carried out using purified Hsp70 in the presence of in concentrations indicated.

Supplementary Figure S2

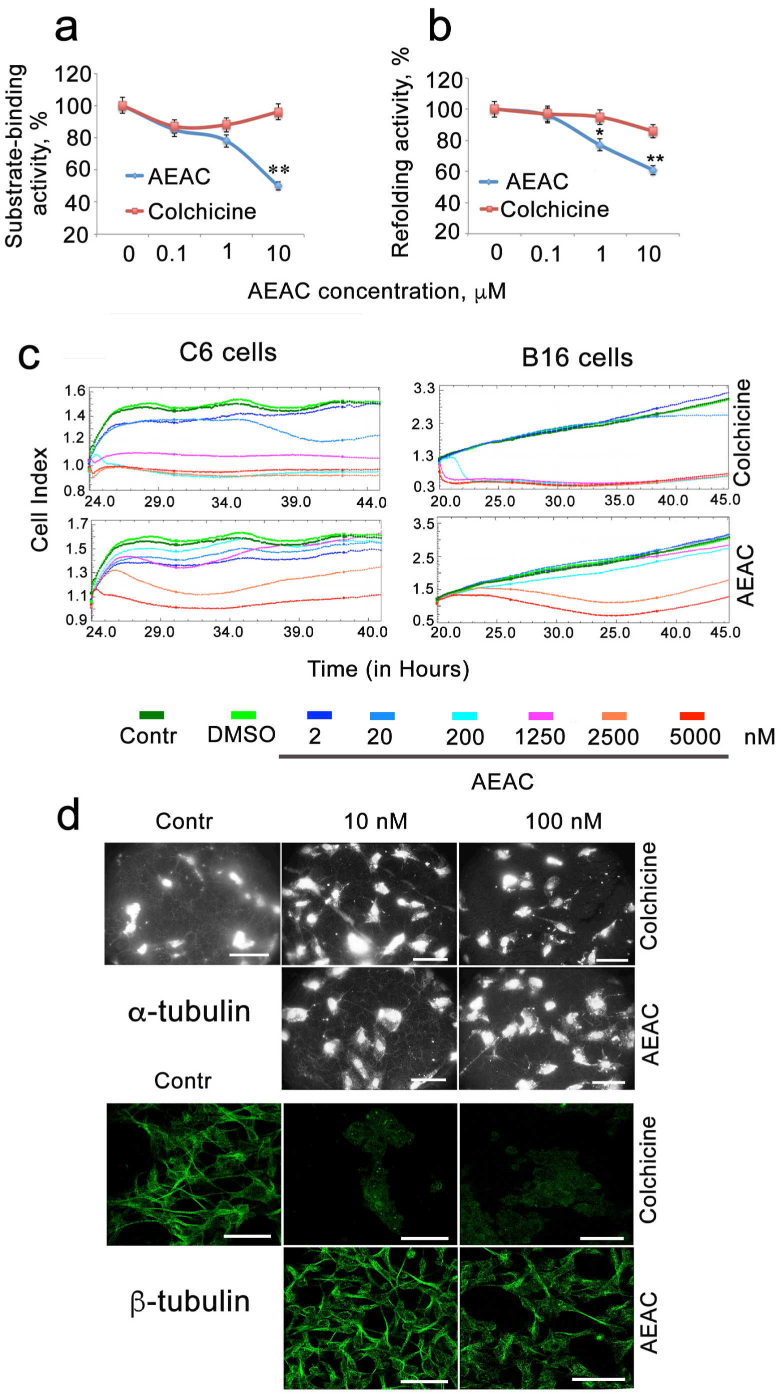

Supplementary Figure S2. The synthetic derivative of colchicine, AEAC, is more effective in the ability to inactivate Hsp70 chaperone proteins compared to colchicine, and is less harmful to cell viability and tubulin disruption.

(A) Substrate-binding and (B) refolding assays carried out using purified Hsp70 in the presence of colchicine or AEAC in concentrations indicated; (C) Proliferation rates of C6 or B16 cells

incubated with AEAC or colchicine. The data are shown as cell index graphs; (D) Comparison of the effects of colchicine and AEAC on tubulin disruption using two approaches.

The upper panel shows monkey kidney Vero cells, which are commonly used for these types of assays, (Zhapparova et al., 2007) were grown on cover slips pretreated with poly-L-lysine. After washing with PBS, 2 μg/ml of nocodazole was added for 3 hours; cells were washed with ice-cold PBS containing 1 mM phenyl-methylsulfonyl fluoride and 10 μg/ml leupeptin; and cells were treated for three minutes with a PEM solution (80 mM PIPES, pH 6.7, 20 mM KCl, 1 mM MgCl2, 1 mM EGTA, 0.1 mM EDTA) that contained 0.5% Triton X-100 and a complete protease inhibitor cocktail (Roche Applied Science, USA). Permeabilized cells (cell "ghosts") were incubated in the PEM solution with 1.2 mg/ml tubulin and 1 mM GTP in the presence of colchicine or AEAC in different concentrations for 40 min at 37°C. The cells were then washed with PEM and fixed in 0.5% glutaraldehyde. FITC-conjugated monoclonal anti-α-tubulin antibodies (Sigma, USA) were used for immunofluorescent staining. Cells with a-tubulin microtubules were visualized using an Axiophot microscope (Zeiss, Germany) equipped with Planaro 63× and 40× objectives and CCD MicroMax camera (Princeton Instruments, USA).

The lower panel shows rat glioma C6 cells that were seeded to cover slips and incubated with colchicine or AEAC for 24 hours. Next, cells were fixed with 4% paraformaldehyde, permeabilized with 0.1% Triton X-100, and stained with anti-β-tubulin antibody (Abcam, UK) followed secondary anti-rabbit antibody conjugated with Alexa488 (Abcam, UK). Cells were examined using a Leica TCS SP2 confocal microscope (Leica, Germany).

Scale bars: 20 μm.

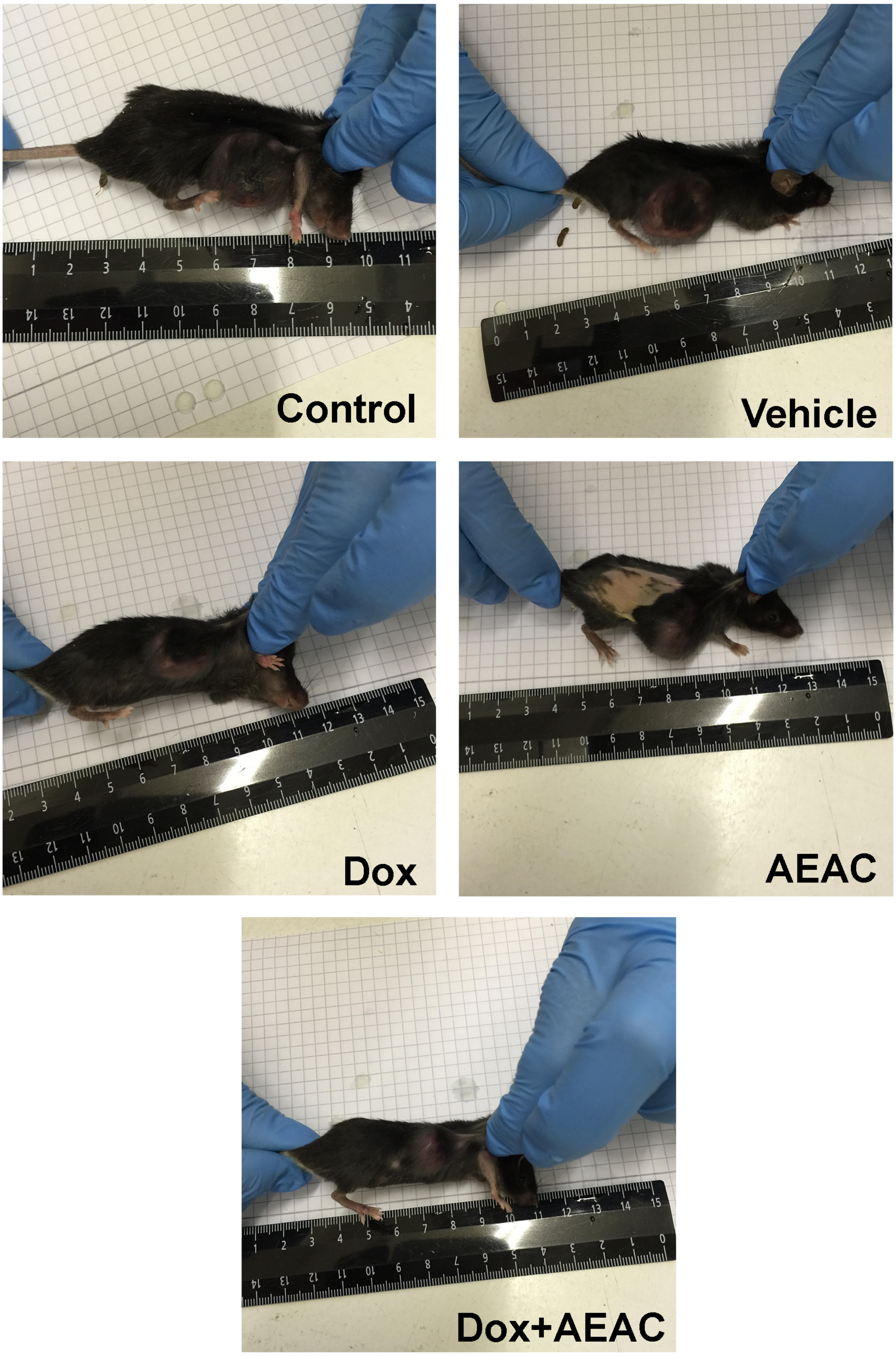

Figure S3. Photos of mice on the 20th day after transplantation of B16 cells.
